# Trends in Early-Onset Colorectal Cancer in Singapore: Epidemiological Study of a Multiethnic Population

**DOI:** 10.2196/62835

**Published:** 2025-02-14

**Authors:** Hui Lionel Raphael Chen, Qingqing Dawn Chong, Brenda Tay, Siqin Zhou, Evelyn Yi Ting Wong, Isaac Seow-En, Ker Kan Tan, Yi Wang, Adeline Seow, Kwong-Wei Emile Tan, Bee Huat Iain Tan, Sze Huey Tan

**Affiliations:** 1 Department of Colorectal Surgery Singapore General Hospital Singapore Singapore; 2 Duke-NUS Medical School Singapore Singapore; 3 Division of Medical Oncology National Cancer Centre Singapore Singapore; 4 Division of Clinical Trials and Epidemiological Sciences National Cancer Centre Singapore Singapore; 5 Division of Colorectal Surgery, Department of Surgery National University Hospital Singapore Singapore; 6 Saw Swee Hock School of Public Health National University of Singapore Singapore Singapore

**Keywords:** early-onset colorectal cancer, epidemiology, Singapore, joinpoint regression, age-period-cohort, public health, health disparity

## Abstract

**Background:**

Colorectal cancer (CRC) incidence and mortality in those aged 50 years and above have decreased over the past 2 decades. However, there is a rising incidence of CRC among individuals under 50 years of age, termed early-onset colorectal cancer (EOCRC). Patients with EOCRC are diagnosed at an advanced stage and may be in more psychosocial, emotional, and financial distress.

**Objective:**

Our study examined the epidemiological shifts in CRC in Singapore, a multiethnic country.

**Methods:**

CRCs diagnosed at age 20 years and above were identified from the Singapore Cancer Registry (SCR) from 1968 to 2019. Patient characteristics included gender, ethnicity, and age of CRC diagnosis. Population information was obtained from the Department of Statistics Singapore (SingStat). Age-specific incidence rates (ASRs) and age-standardized incidence rates (ASIRs) were calculated. The cohort was divided into 3 age groups: 20-49, 50-64, and ≥65 years. Temporal trends in incidence rates were modeled with joinpoint regression. Birth cohort models were fitted using the National Cancer Institute (NCI) age-period-cohort analysis tool. Cancer-specific survival analysis was performed with the Cox proportional hazards model.

**Results:**

In total, 53,044 CRCs were included, and 6183 (11.7%) adults aged 20-49 years were diagnosed with EOCRC. The ASR of EOCRC rose from 5 per 100,000 population in 1968 to 9 per 100,000 population in 1996 at 2.1% annually and rose to 10 per 100,000 population in 2019 at 0.64% annually. The ASR for CRC among adults aged 50-64 years rose at 3% annually from 1968 to 1987 and plateaued from 1987, while the ASR for adults aged 65 years and above rose at 4.1% annually from 1968 to 1989 and 1.3% annually from 1989 to 2003 but decreased from 2003 onwards at 1% annually. The ASR of early-onset rectal cancer increased significantly at 1.5% annually. There was a continued rise in the ASR of EOCRC among males (annual percentage change [APC] 1.5%) compared to females (APC 0.41%). Compared to the 1950-1954 reference birth cohort, the 1970-1984 birth cohort had a significantly higher incidence rate ratio (IRR) of 1.17-1.36 for rectal cancer, while there was no significant change for colon cancer in later cohorts. There were differences in CRC trends across the 3 ethnic groups: Malays had a rapid and persistent rise in the ASR of CRC across all age groups (APC 1.4%-3%), while among young Chinese, only the ASR of rectal cancer was increasing (APC 1.5%). Patients with EOCRC had better survival compared to patients diagnosed at 65 years and above (hazard ratio [HR] 0.73, 95% CI 0.67-0.79, *P*<.001) after adjusting for covariates.

**Conclusions:**

The rise in the incidence of rectal cancer among young adults, especially among Chinese and Malays, in Singapore highlights the need for further research to diagnose CRC earlier and reduce cancer-related morbidity and mortality.

## Introduction

Colorectal cancer (CRC) presents a significant health care problem as it is the third-most common cancer diagnosed and the second-leading cause of death worldwide. In 2020, CRC accounted for 10% of the worldwide cancer incidence and 9% of cancer deaths. Currently, 2 million new cases are diagnosed annually worldwide, which is projected to rise to 3.2 million per annum by 2040 [1,2].

With the implementation of population-wide, average risk CRC screening for all adults aged 50 years or above, CRC incidence and mortality in this age group have progressively decreased over the past 2 decades. However, there is emerging evidence that the incidence of CRC in people under 50 years of age is rising, especially in high-income countries. Based on the World Health Organization–International Agency of Research on Cancer Incidence in Five Continents Plus database, the incidence of early-onset colorectal cancer (EOCRC) rose in 27 (54%) of 50 countries, with the highest incidence rates of 14-16 per 100,000 person-years seen in the United States, Australia, New Zealand, and South Korea [3]. In United States, since the 1990s, colon and rectal cancer incidence has been increasing at 1.3% and 2.3% per year, respectively, for adults aged 40-49 years [4]. This is in contrast to the declining incidence of CRC among adults aged 55 years or older. Similar trends in colon and rectal cancer incidence were observed in Australia, with an increase of 1.7% and 0.9% per year, respectively, for adults aged 40-49 years [5].

EOCRC may be associated with hereditary syndromes, such as Lynch syndrome and familial adenomatous polyposis, but these only account for 1%-3% of the total number of new CRC cases. The large majority of EOCRC cases are still sporadic and contribute 10%-12% of new CRC cases. EOCRC is more likely to occur in the left colon, with higher rates of mucinous and poorly differentiated cancers, which may be associated with poor prognosis [6]. Patients with EOCRC tend to possess an underappreciation of symptoms; more reluctance to seek medical assistance, leading to delayed diagnosis; and more advanced stage at diagnosis [7]. They present with unique challenges—as younger patients with young children, heavy financial commitments, and fertility preservation [8]. The rise in the incidence of EOCRC may be a consequence of increased endoscopic capacity, which may lower the threshold for performing colonoscopy in younger patients. However, another important factor is the strong birth cohort effect observed worldwide, which may be the result of changes in exposure to risk factors. Although most studies have found EOCRC prevalence to be independent of gender, racial disparities in EOCRC incidence have been described in the United States, with a higher incidence seen in African Americans [9,10].

Singapore has a multiethnic resident population of 4 million, of which 74% are Chinese, 13% Malays, and 9% Indians [11]. In Singapore, CRC has the highest incidence among all cancers for the past several years, which, therefore, imposes a substantial burden in terms of complications, mortality, health care resources, and medical costs [12]. According to the *Singapore Cancer Registry Annual Report 2021*, there were a total of 12,239 new cases of CRC diagnosed from 2017 to 2021. There was a notable rise in the age-standardized incidence rate (ASIR) of CRC from 19.4 per 100,000 population in 1968-1972 to 37.5 per 100,000 population in 2017-2021 among men. A similar trend among females was observed, at 15.4 per 100,000 population in 1968-1972 to 26.9 per 100,000 population in 2017-2021. Among those aged 40-49 years, CRC was the most common cause of cancer among men and the fourth-most common cause of cancer among women. There were differing trends in the ASIRs of cancer among the 3 main ethnic groups. Although the Chinese had the highest ASIR of cancer throughout the years, there is evidence of a closing ethnic gap in cancer incidence between the Chinese and Malays [13]. Although there has been an overall increase in survival rates for CRC over the past decades, the age-specific patterns and effects of gender and ethnicity on the incidence trends in CRC in Singapore are not well studied. Therefore, the aim of this study was to perform a comprehensive analysis of temporal trends in colon and rectal cancer incidence in Singapore.

## Methods

### Study Design and Data Source

The Singapore Cancer Registry (SCR) is a population-based registry that was established in 1968 and is part of the National Registry of Disease Office. The SCR collects notifications from medical practitioners about all patients with cancer diagnosed in Singapore, as mandated by the National Registry of Diseases Act 2007. The data are internationally comparable and valid, with completeness of about 97.5%. Annual audits of the data are performed to ensure data accuracy of at least 95% as the data are critical to guide cancer prevention policies and cancer research [14].

Patients aged 20 years and older diagnosed with colon cancer (*International Classification of Diseases* [ICD]-9 code 153, ICD-10 code C18) and rectal cancer (ICD-9 codes 154.0, 154.1; ICD-10 codes C19.9, C20.9) from 1968 to 2019 from the SCR were included in this study. Recurrent and metachronous CRCs, premalignant tumors, carcinoma in situ, extranodal colorectal lymphoma, and sarcomas were excluded. Information about the Singapore population was obtained from the Department of Statistics Singapore (SingStat), which provides publicly available data that were used to define the denominator for incidence rates [11]. Patient characteristics included gender, ethnicity, and age at diagnosis. The tumor, node, and metastasis (TNM) stage at diagnosis was available for patients diagnosed from 2003 onward.

Colon cancers were separated from cancers of the rectosigmoid and rectum, which were grouped together as rectal cancer for analysis. Age-specific incidence rates (ASRs) were calculated as the number of new CRC cases diagnosed each year divided by the population at risk for that age group. The population at risk was defined as the group of individuals susceptible to CRC during the period of interest. ASIRs were calculated as the sum of weighted ASRs using the direct method and the Segi-Doll World Standard as the reference population.

### Joinpoint Analysis

The cohort was initially divided into 3 age groups: 20-49, 50-64, and ≥65 years. EOCRC was defined as CRC diagnosed at age 20-49 years. Annual incidence rates from 1968 to 2019 were estimated for the 3 age groups. Joinpoint regression analysis was used to describe the temporal trends in cancer incidence, identify points of change in direction, and estimate the magnitude of the change in rates. Joinpoint analysis estimates the annual percentage change (APC) by assessing observed data and fitting a series of joined straight lines to determine the APC over each period. The changes are tested for statistical significance [15,16]. It assumes that the regression function is piecewise linear, and the segments are continuously connected at unknown change points [17]. Compared to the traditional Monte Carlo permutation method, the data-driven weighted Bayes information criterion (BIC) was used for model selection as it was computationally more efficient yet produced similar results [18].

### Age-Period-Cohort Modeling

Birth cohort models were fitted using the National Cancer Institute (NCI) Age Period Cohort web tool [19]. Age-period-cohort modeling provides estimates of parameters that describe relationships between observed incidence rates and age, calendar period, and birth cohort based on age groups and time periods of equal length. There was no information available for those aged ≥70 years for several time points from SingStat population estimates. Therefore, age-period-cohort analysis was limited to those aged 20-69 years. Input data were CRC cases and population counts for 10 five-year time periods (1970-1974, 1975-1979, …, 2015-2019), 10 five-year age groups (20-24, 25-29, …, 65-69 years), and 19 birth cohorts generated starting from the 1905-1909 to the 1995-1999 birth cohort. Cohort effects were presented as incidence rate ratios (IRRs) for a given birth cohort, with the 1950-1954 as the reference birth cohort. Additionally, local drifts estimated the age-specific net APC in incidence rates across the different age groups.

### Survival Analysis

Cancer-specific survival was defined as the period from the date of CRC diagnosis to the date of death due to CRC. Patients who died of other causes were censored at their date of death; similarly, patients who were still alive were censored at the date of last follow-up. Clinical characteristics, such as surgical, radiation, and chemotherapy treatments received, were available. Multivariable survival analysis was performed with the Cox proportional hazards model.

### Ethical Considerations

The study was approved by the SingHealth Institutional Review Board (IRB 2022/2415) to access and analyze data from the SCR. Informed consent was waived (waiver of consent was approved by the SingHealth IRB) as study data were deidentified.

## Results

### Characteristics of the Study Population

**Figure 1:**
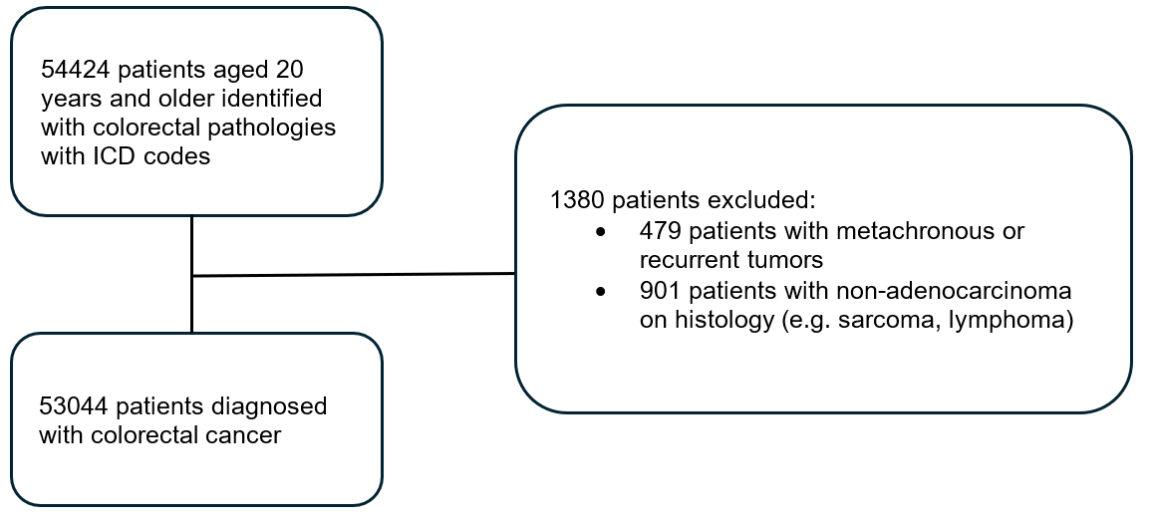
Flowchart of the patients identified and extracted from the SCR. ICD: *International Classification of Diseases*; SCR: Singapore Cancer Registry.

In total, 53,044 CRCs were included, with 32,880 (62%) colon and 20,164 (38%) rectal cancers. Figure 1 describes the flowchart of identifying and extracting patients from the SCR. Furthermore, 6183 (11.7%) EOCRCs were diagnosed, with 3585 (58%) colon and 2598 (42%) rectal cancers. Clinical characteristics of the study population are described in Table 1. There was a higher proportion of stage 3 (37.4% vs 32.4%) and stage 4 (26.1% vs 24%) CRCs and a lower proportion of stage 2 (21.2% vs 28.1%) CRCs among EOCRCs compared to patients diagnosed at an age of 65 years and above (*P*<.001).

**Table 1:** Characteristics of CRCs^a^ from the SCR^b^.

Characteristics		Total cases (N=53,044), n (%)	Group 1 (20-49 years; n=6183), n (%)	Group 2 (50-64 years; n=17,862), n (%)	Group 3 (≥65 years; n=28,999), n (%)
**Gender; *P*<.001**					
	Male	28,429 (53.6)	3235 (52.3)	10,245 (57.4)	14,949 (51.6)
	Female	24,615 (46.4)	2948 (47.7)	7617 (42.6)	14,050 (48.4)
**Ethnicity; *P*<.001**					
	Chinese	46,831 (88.3)	5167 (83.6)	15,495 (86.7)	26,169 (90.2)
	Indian	1767 (3.3)	305 (4.9)	671 (3.8)	791 (2.7)
	Malay	3723 (7.0)	583 (9.4)	1448 (8.1)	1692 (5.8)
	Others	723 (1.4)	128 (2.1)	248 (1.4)	347 (1.2)
**Tumor site; *P*<.001**					
	Colon	32,880 (62)	3585 (58.0)	10,332 (57.8)	18,963 (65.4)
	Rectum	20,164 (38)	2598 (42.0)	7530 (42.2)	10,036 (34.6)
**TNM^c^ stage (2003 and beyond); *P*<.001**					
	1	4436 (15.9)	397 (15.3)	1643 (16.6)	2396 (15.6)
	2	7178 (25.7)	551 (21.2)	2299 (23.2)	4328 (28.1)
	3	9495 (34)	969 (37.4)	3534 (35.6)	4992 (32.4)
	4	6830 (24.4)	677 (26.1)	2448 (24.7)	3705 (24.0)

^a^CRC: colorectal cancer.

^b^SCR: Singapore Cancer Registry.

^c^TNM: tumor, node, and metastasis.

### Joinpoint Regression Analysis of Incidence Rates of Colorectal, Colon, and Rectal Cancers

**Figure 2:**
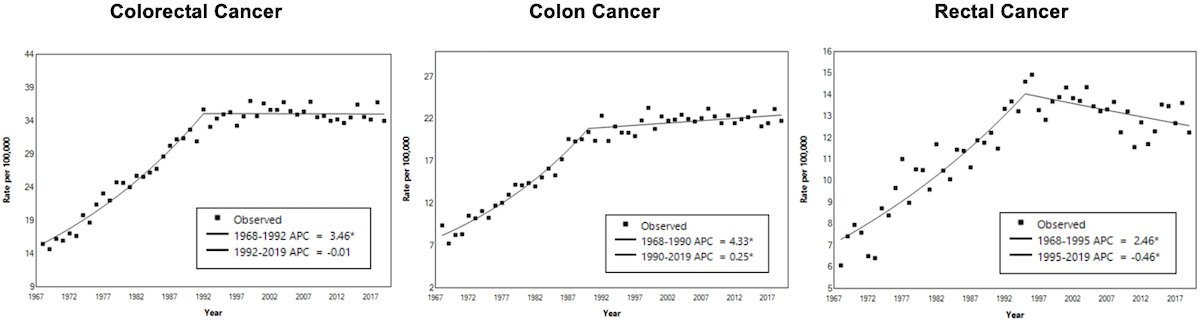
Joinpoint regression of the ASIR of CRC, colon cancer, and rectal cancer. APC: annual percentage change; ASIR: age-standardized incidence rate; CRC: colorectal cancer. See Multimedia Appendix 1 for a high-resolution version.

The ASIR of CRC rose from 15 in 1968 to 36 per 100,000 population in 1992 at an APC of 3.5% and plateaued (Figure 2). The ASIR of colon cancer rose from 8 in 1968 to 20 per 100,000 population in 1990 at an APC of 4.3% and plateaued. The ASIR of rectal cancer rose from 7 in 1968 to 14 per 100,000 population in 1990 at an APC of 2.5% and decreased to 12 per 100,000 population in 2019 at an APC of –0.5%.

**Figure 3:**
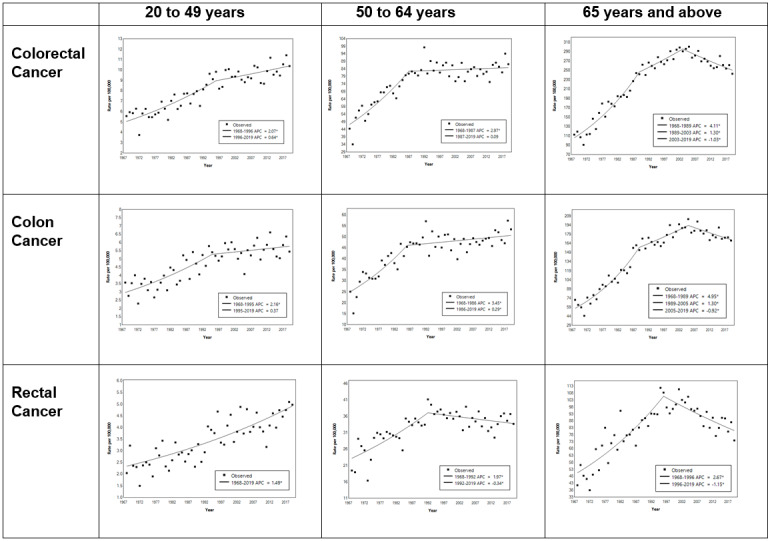
Joinpoint regression of the ASRs of CRC, colon cancer, and rectal cancer by age group and tumor location. APC: annual percentage change; ASR: age-specific incidence rate; CRC: colorectal cancer. See Multimedia Appendix 1 for a high-resolution version.

The results of joinpoint regression are shown in Table 2 and Figure 3 for CRC, colon cancer, and rectal cancer stratified by age group. For EOCRC, the ASR rose from 5 in 1968 to 9 in 1997 at an APC of 2% and increased to 10 per 100,000 population in 2019 at an APC of 0.6%. For those aged 50-64 years, trends were similar to the ASIR of CRC for the overall Singapore population. For those aged 65 years and above, the ASR of CRC rose from 113 in 1968 to 299 in 2002 at an APC of 4.11% and decreased to 242 per 100,000 population in 2019 at an APC of –1.03%.

The ASR of early-onset colon cancer rose from 3 in 1968 to 5 per 100,000 population in 1995 at an APC of 2.2% and thereafter had a nonsignificant increase at an APC of 0.4%. For those aged 50-64 years, colon cancer trends were similar to the ASIR of CRC for the overall Singapore population. For those aged 65 years and above, the ASR of colon cancer rose from 57 in 1968 to 193 in 2005 at an APC of 4.95% and decreased to 170 per 100,000 population in 2019 at an APC of –0.92%.

The ASR of early-onset rectal cancer rose from 2 in 1968 to 5 per 100,000 population in 2019 at an APC of 1.5% per year. For those aged 50-64 years and 65 years and above diagnosed with rectal cancer, trends were similar to the ASIR of rectal cancer for the overall Singapore population (Table 2 and Figure 3).

**Table 2:** APC^a^ of CRC^b^, colon cancer, and rectal cancer ASRs^c^ by age group and tumor location. See Multimedia Appendix 1 for a high-resolution version.

Type of cancer and age groups		Patients, n (%)	Trend 1		Trend 2		Trend 3	
			Years	APC, %	Years	APC, %	Years	APC, %
**CRC**								
	20-49 years	6183 (11.6)	1968-1996	2.07^d^	1996-2019	0.64^d^	—^e^	—
	50-64 years	17,862 (33.7)	1968-1987	2.97^d^	1987-2019	0.09	—	—
	≥65 years	28,999 (54.7)	1968-1989	4.11^d^	1989-2003	1.30^d^	2003-2019	–1.03^f^
**Colon cancer**								
	20-49 years	3585 (10.9)	1968-1995	2.16^d^	1995-2019	0.37	—	—
	50-64 years	10,332 (31.4)	1968-1986	3.45^d^	1986-2019	0.29^d^	—	—
	≥65 years	18,963 (57.7)	1968-1989	4.95^d^	1989-2005	1.30^d^	2005-2019	–0.92^f^
**Rectal cancer**								
	20-49 years	2598 (12.9)	1968-2019	1.49^d^	—	—	—	—
	50-64 years	7530 (37.3)	1968-1992	1.97^d^	1992-2019	–0.34^f^	—	—
	≥65 years	10,036 (49.8)	1968-1996	2.67^d^	1996-2019	–1.15^f^	—	—

^a^APC: annual percentage change.

^b^CRC: colorectal cancer.

^c^ASR: age-specific incidence rate.

^d^Statistically significant positive APCs.

^e^Not applicable.

^f^Statistically significant negative APCs.

### Effect of Gender

**Figure 4:**
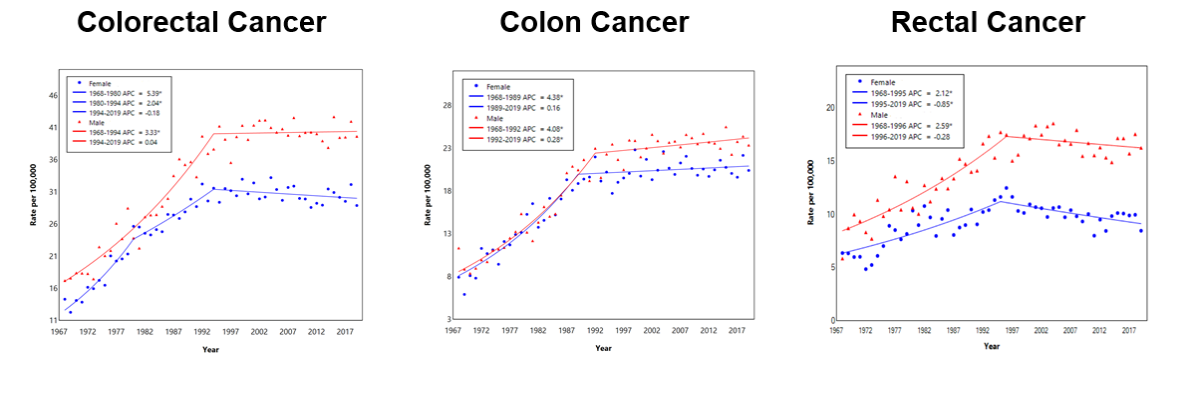
Joinpoint regression of ASRs of CRC, colon cancer, and rectal cancer by gender (male: red; female: blue). APC: annual percentage change; ASR: age-specific incidence rate; CRC: colorectal cancer. See Multimedia Appendix 1 for a high-resolution version.

**Figure 5:**
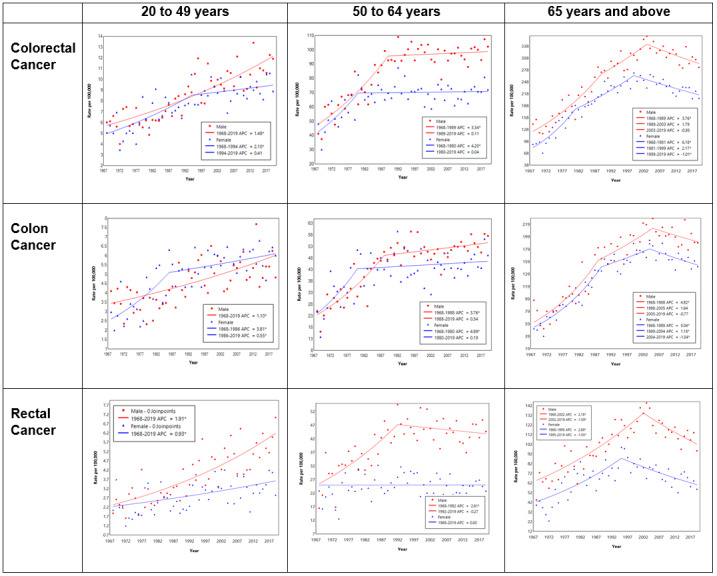
Joinpoint regression of ASRs of CRC, colon cancer, and rectal cancer by age group and gender (male: red; female: blue). APC: annual percentage change; ASR: age-specific incidence rate; CRC: colorectal cancer. See Multimedia Appendix 1 for a high-resolution version.

The male population had higher incidence rates for colon and rectal cancers. Similar to the ASIR, for both genders aged 50-64 years and 65 years and above, the incidence of CRC, colon cancer, and rectal cancer rose before it plateaued or decreased in the recent 2 decades (Table 3 and Figures 4 and 5). Among those aged 50-64 years, there was a rapid initial rise in colon cancer among women compared to men (APC 5% vs 3.8%).

For males aged 20-49 years, there was a rising incidence in CRC (APC 1.5%), mainly rectal cancer (APC 1.9%), with a smaller rise in colon cancer (APC 1.1%). For females aged 20-49 years, similar to older females aged 50-64 years, there was a rapid initial rise, particularly in colon cancer (APC 3.8%); however, in recent years, for colon cancer, rectal cancer, and CRC, the APC has been <1% (Table 3 and Figure 5).

**Table 3:** APC^a^ of CRC^b^, colon cancer, and rectal cancer ASRs^c^ by age group, tumor location, and gender.

Type of cancer and age groups		Patients, n (%)	Trend 1		Trend 2		Trend 3	
			Years	APC, %	Years	APC, %	Years	APC, %
**CRC (20-49 years)**								
	Male	3235 (52.3)	1968-2019	1.48^d^	—^e^	—	—	—
	Female	2948 (47.7)	1968-1994	2.10^d^	1994-2019	0.41	—	—
**CRC (50-64 years)**								
	Male	10,245 (57.4)	1968-1989	3.34^d^	1989-2019	0.11	—	—
	Female	7617 (42.6)	1968-1980	4.20^d^	1980-2019	0.04	—	—
**CRC (≥65 years)**								
	Male	14,949 (51.6)	1968-1989	3.76^d^	1989-2003	1.79	2003-2019	–0.95
	Female	14,050 (48.4)	1968-1981	6.18^d^	1981-1999	2.17^d^	1999-2019	–1.01^f^
**Colon cancer (20-49 years)**								
	Male	1715 (47.8)	1968-2019	1.10^d^	—	—	—	—
	Female	1870 (52.2)	1968-1986	3.81^d^	1986-2019	0.55^d^	—	—
**Colon cancer (50-64 years)**								
	Male	5471 (53.0)	1968-1988	3.76^d^	1988-2019	0.34	—	—
	Female	4861 (47.0)	1968-1980	4.99^d^	1980-2019	0.19	—	—
**Colon cancer (≥65 years)**								
	Male	9289 (49.0)	1968-1988	4.92^d^	1988-2005	1.64	2005-2019	–0.77
	Female	9674 (51.0)	1968-1989	5.04^d^	1989-2004	1.18^d^	2004-2019	–1.04^f^
**Rectal cancer (20-49 years)**								
	Male	1520 (58.5)	1968-2019	1.91^d^	—	—	—	—
	Female	1078 (41.5)	1968-2019	0.93^d^	—	—	—	—
**Rectal cancer (50-64 years)**								
	Male	4774 (63.4)	1968-1989	2.61^d^	1992-2019	–0.27	—	—
	Female	2756 (36.6)	1968-2019	0	—	—	—	—
**Rectal cancer (≥65 years)**								
	Male	5660 (56.4)	1968-2002	2.18^d^	2002-2019	–1.59^f^	—	—
	Female	4376 (43.6)	1968-1995	2.69^d^	1995-2004	–1.55^f^	—	—

^a^APC: annual percentage change.

^b^CRC: colorectal cancer.

^c^ASR: age-specific incidence rate.

^d^Statistically significant positive APCs.

^e^Not applicable.

^f^Statistically significant negative APCs.

### Effect of Ethnicity

**Figure 6:**
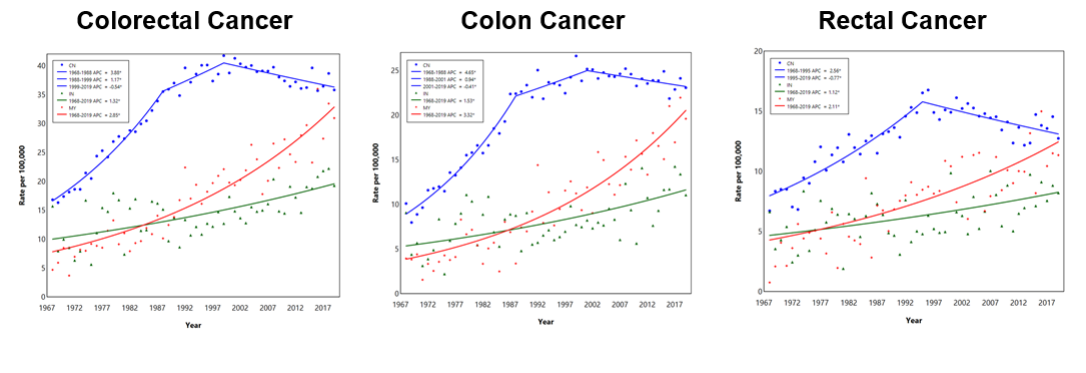
Joinpoint regression of ASIRs of CRC, colon cancer, and rectal cancer by ethnic group (Chinese/CN: blue; Indian/IN: green; Malay/MY: red). APC: annual percentage change; ASIR: age-standardized incidence rate; CRC: colorectal cancer. See Multimedia Appendix 1 for a high-resolution version.

**Figure 7:**
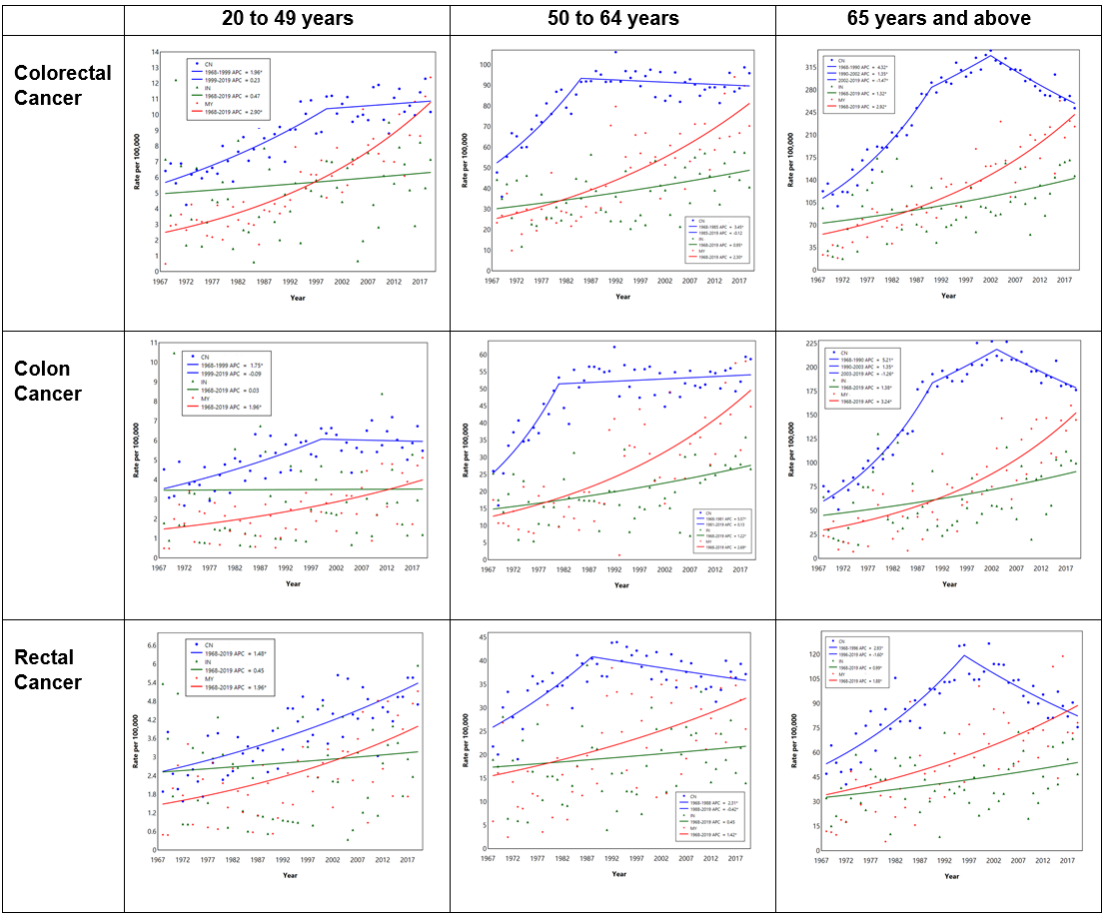
Joinpoint regression of ASRs of CEC, colon cancer, and rectal cancer by age group, tumor location, and ethnic group (Chinese/CN: blue; Indian/IN: green; Malay/MY: red). APC: annual percentage change; ASR: age-specific incidence rate; CRC: colorectal cancer. See Multimedia Appendix 1 for a high-resolution version.

Similar to the ASIR, for Chinese patients aged 50-64 years and 65 years and above, the ASR of CRC, colon cancer, and rectal cancer showed an initial rise but plateaued or decreased in the recent 2 decades. In Malays across all 3 age groups, there was a persistent rise in the ASR of CRC, colon cancer, and rectal cancer (APC 1.4%-3.2%) without evidence of it plateauing. In Indians aged 50-64 years and 65 years and above, there was a smaller persistent rise in the ASR of CRC, colon cancer, and rectal cancer (APC 1%-1.4%) without evidence of it plateauing (Tables 4-6 and Figures 6 and 7).

**Table 4:** APC^a^ of CRC^b^, colon cancer, and rectal cancer ASRs^c^ for patients diagnosed at age 20-49 years by tumor location and ethnicity.

Tumor location and ethnicity		Patients, n (%)	Trend 1		Trend 2		Trend 3	
			Years	APC, %	Years	APC, %	Years	APC, %
**CRC**								
	Chinese	5167 (85.3)	1968-1999	1.96^d^	1999-2019	0.23	—^e^	—
	Indian	305 (5.1)	1968-2019	0.47	—	—	—	—
	Malay	583 (9.6)	1968-2019	2.90^d^	—	—	—	—
**Colon cancer**								
	Chinese	2992 (85.4)	1968-1999	1.75^d^	1999-2019	–0.09	—	—
	Indian	164 (4.7)	1968-2019	0.03	—	—	—	—
	Malay	346 (9.9)	1968-2019	1.96^d^	—	—	—	—
**Rectal cancer**								
	Chinese	2175 (85.2)	1968-2019	1.48^d^	—	—	—	—
	Indian	141 (5.5)	1968-2019	0.45	—	—	—	—
	Malay	237 (9.3)	1968-2019	1.96^d^	—	—	—	—

^a^APC: annual percentage change.

^b^CRC: colorectal cancer.

^c^ASR: age-specific incidence rate.

^d^Statistically significant positive APCs.

^e^Not applicable.

**Table 5:** APC^a^ of CRC^b^, colon cancer, and rectal cancer ASRs^c^ for patients diagnosed at age 50-64 years by tumor location and ethnicity.

Tumor location and ethnicity		Patients, n (%)	Trend 1		Trend 2		Trend 3	
			Years	APC, %	Years	APC, %	Years	APC, %
**CRC**								
	Chinese	15,495 (88.0)	1968-1985	3.45^d^	1985-2019	–0.12	—^e^	—
	Indian	671 (3.8)	1968-2019	0.95^d^	—	—	—	—
	Malay	1448 (8.2)	1968-2019	2.30^d^	—	—	—	—
**Colon cancer**								
	Chinese	9012 (88.4)	1968-1981	5.57^d^	1981-2019	0.13	—	—
	Indian	356 (3.5)	1968-2019	1.22^d^	—	—	—	—
	Malay	823 (8.1)	1968-2019	2.69^d^	—	—	—	—
**Rectal cancer**								
	Chinese	6483 (87.3)	1968-1988	2.31^d^	1988-2019	–0.42^f^	—	—
	Indian	315 (4.3)	1968-2019	0.45	—	—	—	—
	Malay	625 (8.4)	1968-2019	1.42^d^	—	—	—	—

^a^APC: annual percentage change.

^b^CRC: colorectal cancer.

^c^ASR: age-specific incidence rate.

^d^Statistically significant positive APCs.

^e^Not applicable.

^f^Statistically significant negative APCs.

**Table 6:** APC^a^ of CRC^b^, colon cancer, and rectal cancer ASRs^c^ for patients diagnosed at age ≥65 years by tumor location and ethnicity.

Tumor location and ethnicity		Patients, n (%)	Trend 1		Trend 2		Trend 3	
			Years	APC, %	Years	APC, %	Years	APC, %
**CRC**								
	Chinese	26,169 (91.3)	1968-1990	4.32^d^	1990-2002	1.35^d^	2002-2019	–1.47^e^
	Indian	791 (2.8)	1968-2019	1.32^d^	—^f^	—	—	—
	Malay	1692 (5.9)	1968-2019	2.97^d^	—	—	—	—
**Colon cancer**								
	Chinese	17,254 (92.0)	1968-1990	5.21^d^	1990-2003	1.35^d^	2003-2019	–1.26^e^
	Indian	483 (2.6)	1968-2019	1.38^d^	—	—	—	—
	Malay	1006 (5.4)	1968-2019	3.24^d^	—	—	—	—
**Rectal cancer**								
	Chinese	8915 (90.0)	1968-1996	2.93^d^	1996-2019	–1.60^e^	—	—
	Indian	308 (3.1)	1968-2019	0.99^d^	—	—	—	—
	Malay	686 (6.9)	1968-2019	1.88^d^	—	—	—	—

^a^APC: annual percentage change.

^b^CRC: colorectal cancer.

^c^ASR: age-specific incidence rate.

^d^Statistically significant positive APCs.

^e^Statistically significant negative APCs.

^f^Not applicable.

Malay patients aged 20-49 years had the highest rise in the ASR of CRC (APC 2.9%), colon cancer (APC 2%), and rectal cancer (APC 2%). Among Chinese patients aged 20-49 years, the ASR of rectal cancer persistently rose (APC 1.5%), while the ASR of CRC and colon cancer plateaued from 1999. In Indians aged 20-49 years, there was a small and nonsignificant rise in the ASR of CRC (APC 0.5%) and rectal cancer (APC 0.5%), as shown in Tables 4-6 and Figure 7.

### Age-Period-Cohort Model of IRRs and APCs for CRC, Colon Cancer, and Rectal Cancer

**Figure 8:**
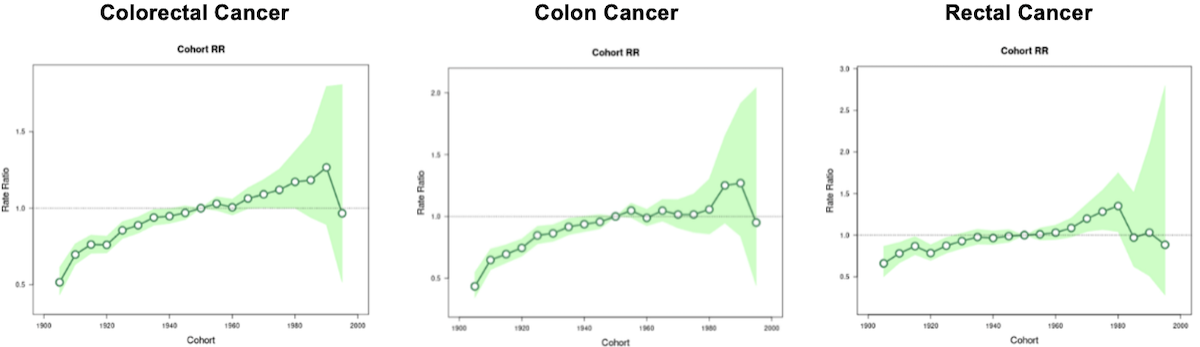
IRRs by birth cohort for CRC, colon cancer, and rectal cancer. CRC: colorectal cancer; IRR: incidence rate ratio. See Multimedia Appendix 1 for a high-resolution version.

Table 7 and Figure 8 show the IRRs by birth cohort for CRC, colon cancer, and rectal cancer. Compared to the reference birth cohort of 1950-1954, there was a trend toward higher IRRs for colon cancer among the 1970-1990 birth cohorts; however, these changes were not statistically significant. In addition, compared to the reference birth cohort of 1950-1954, the IRRs for rectal cancer among the birth cohorts from 1905 onward were marginally lower. However, there was significant increase in the IRR for rectal cancer among the birth cohorts from 1970 onward, and this peaked in the 1980-1984 birth cohort (IRR 1.35, 95% CI 1.043-1.748). Subsequent birth cohorts from 1985 onward had similar IRRs as the reference birth cohort (Figure 7 and Table 7).

**Figure 9:**
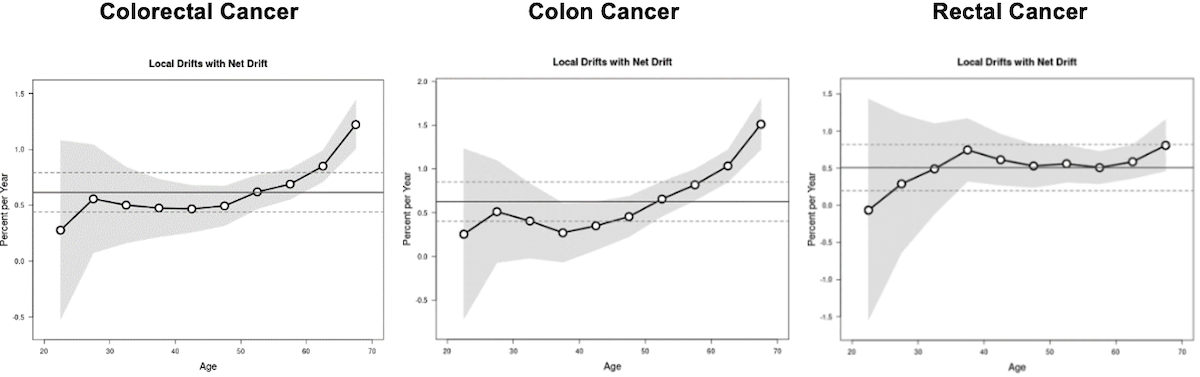
Age-specific APC (local drift) for CRC, colon cancer, and rectal cancer. APC: annual percentage change; CRC: colorectal cancer. See Multimedia Appendix 1 for a high-resolution version.

Figure 9 shows the net age-specific APC or local drift for CRC, colon cancer, and rectal cancer incidence rates. There was an increase in the incidence rates for CRC among those aged 25-69 years, with an average APC of 0.61% (95% CI 0.44-0.79) for all age groups over the entire period. There was an increase in incidence rates for colon cancer among those aged 40-69 years, while there was an increase in incidence rates for rectal cancer among those aged 35-69 years.

**Table 7:** IRRs^a^ for CRC^b^, colon cancer, and rectal cancer by birth cohort.

Birth cohort	CRC IRR (95% CI)	Colon cancer IRR (95% CI)	Rectal cancer IRR (95% CI)
1905-1909	0.516 (0.435-0.613)	0.434 (0.345-0.547)	0.660 (0.503-0.867)
1910-1914	0.697 (0.633-0.768)	0.648 (0.570-0.736)	0.781 (0.668-0.912)
1915-1919	0.763 (0.706-0.825)	0.696 (0.626-0.771)	0.867 (0.765-0.983)
1920-1924	0.761 (0.708-0.817)	0.745 (0.679-0.817)	0.784 (0.696-0.883)
1925-1929	0.856 (0.802-0.912)	0.846 (0.779-0.920)	0.873 (0.784-0.972)
1930-1934	0.889 (0.837-0.943)	0.863 (0.799-0.933)	0.929 (0.842-1.025)
1935-1939	0.939 (0.889-0.993)	0.916 (0.852-0.984)	0.977 (0.892-1.070)
1940-1944	0.948 (0.901-0.997)	0.937 (0.878-1.000)	0.966 (0.889-1.050)
1945-1949	0.970 (0.926-1.015)	0.956 (0.901-1.015)	0.988 (0.915-1.067)
1950-1954	1.000 (1.000)	1.000 (1.000)	1.000 (1.000)
1955-1959	1.030 (0.986-1.075	1.047 (0.991-1.107)	1.010 (0.940-1.086)
1960-1964	1.006 (0.956-1.059) )	0.989 (0.925-1.057)	1.030 (0.947-1.120)
1965-1969	1.063 (0.998-1.133)	1.047 (0.964-1.137)	1.086 (0.978-1.205)
1970-1974	1.091 (1.002-1.187)	1.015 (0.907-1.136)	1.199 (1.045-1.375)
1975-1979	1.120 (0.999-1.255)	1.016 (0.873-1.182)	1.28 (1.067-1.541)
1980-1984	1.172 (1.001-1.372)	1.057 (0.858-1.302)	1.35 (1.043-1.748)
1985-1989	1.184 (0.940-1.491)	1.251 (0.947-1.652)	0.971 (0.623-1.512)
1990-1994	1.266 (0.893-1.795)	1.269 (0.841-1.914)	1.031 (0.511-2.080)
1995-1999	0.967 (0.517-1.807)	0.951 (0.443-2.040)	0.884 (0.280-2.792)

^a^IRR: incidence rate ratio.

^b^CRC: colorectal cancer.

### Survival Outcomes

Patients with EOCRC had better cancer-specific survival compared to patients diagnosed at 65 years and above (hazard ratio [HR] 0.73, 95% CI 0.67-0.79, *P*<.001) after adjusting for the TNM stage, gender, tumor site, surgery, radiotherapy, and chemotherapy (Table 8).

**Table 8:** Multivariable cancer-specific survival analysis of patients with CRC^a^ using the Cox proportional hazards model.

Variables and groups			HR^b^ (95% CI)	*P* value
**Age (years)**				
	20-49 vs ≥65	0.73 (0.67-0.79)		<.001
	50-64 vs ≥65	0.74 (0.70-0.78)		<.001
**TMN^c^ stage**				
	2 vs 1	3.84 (3.23-4.56)		<.001
	3 vs 1	11.14 (9.43-13.16)		<.001
	4 vs 1	58.70 (49.66-69.38)		<.001
**Gender: male vs female**			1.06 (1.02-1.11)	.006
**Tumor site: rectum vs colon**			0.55 (0.52-0.58)	<.001
**Treatment**				
	Surgery vs no surgery	0.43 (0.41-0.46)		<.001
	Radiotherapy vs no radiotherapy	1.10 (1.01-1.19)		<.026
	Chemotherapy vs no chemotherapy	0.54 (0.51-0.57)		<.001

^a^CRC: colorectal cancer.

^b^HR: hazard ratio.

^c^TNM: tumor, node, and metastasis.

## Discussion

### Principal Findings

Historically, CRC has been more commonly associated with older age groups, but in recent years, many countries have reported a concerning rise in cases among younger individuals. Although the overall incidence of CRC has been declining in older people, there has been a notable increase in the incidence of EOCRC, particularly in individuals younger than 50 years. In the United States, the overall annual, CRC ASIR has decreased by 46% from 66.2 per 100,000 population in 1985 to 35.7 per 100,000 population in 2019. Notably, CRC is now the most common cause of cancer deaths among individuals less than 50 years old [20]. However, from 2011 through 2019, the CRC incidence rate increased by 1.9% per year for individuals younger than 50 years. Rectal cancer has driven the EOCRC epidemiological trend with an increase of 2% per year. There was a steep decline in incidence among adults aged 50 years and older in the United States at 3%-5% annually in the late 2000s, and this is likely attributed to changing patterns in the risk factors and uptake of CRC screening. Incidence rates have been increasing in adults aged 20-39 years since the mid-1980s and in those aged 40-54 years since the mid-1990s [21]. Similar trends are seen in Canada, the United Kingdom, Australia, New Zealand, Europe, and Asia [5,22-25]. The rising incidence of EOCRC in East Asia may be due to an increase in the prevalence of risk factors, such as smoking, alcoholism, and obesity among young individuals [26]. A population-based study in Taiwan, Korea, Japan, and Hong Kong suggests that from 1995 to 2014, the pooled incidence of early-onset colon and rectal cancers increased by 2.1% and 3.8%, respectively, among men. Similarly, the pooled incidence of early-onset colon and rectal cancers increased by 2.2% and 4%, respectively, among women [27]. In China, the incidence rate of EOCRC rose from 3.6 to 12.1 per 100,000 population from 1990 to 2019 at a rate of 4.6% per year [25].

Our analysis of incidence trends over time suggests that the rising incidence of EOCRC in Singapore is nuanced. The rising trend for people under 50 years of age is particularly for rectal cancer, especially in males. This trend also affects, in particular, the Malays but also the Chinese, with the Indians less affected. Our study supports the increasing literature that the increase in EOCRC is not confined to the West and may be a worldwide phenomenon. The cause of these observations is still unclear, but it can be explained by the cohort effects. The IRR for rectal cancer has been increasing among the 1970-1984 birth cohorts and suggests that it is the changes in exposure to these birth cohorts that influence the risk of carcinogenesis. High consumption of processed meat, alcohol, a sedentary lifestyle, a lack of dietary fiber consumption, smoking, and obesity are known lifestyle factors associated with CRC. However, these modifiable risk factors are present in all age groups and not specific to young individuals and cannot explain fully the rising incidence among young people [28,29]. It has also been reported that there are similar rates of obesity between EOCRC and older patients with CRC [30]. The increasing prevalence of the use of antibiotics and alterations in the gut microbiome may play a role in the development of CRC through effects of bacterial-derived metabolites and virulence factors. Results of a recent study show an increase in *Fusobacterium* spp. in the microbiome of EOCRC [31]. Understanding the generational shifts in early life exposures may provide further insights into the etiologies of EOCRC [7].

Most tumors in EOCRC are in the sigmoid colon and rectum [32]. The reasons regarding the steep rise in the incidence of rectal compared to colon cancer among young adults are unclear. Understanding the differences in the molecular profile between rectal and colon cancers may be key toward developing preventative strategies. EOCRC in young adults has been associated with synchronous and metachronous tumors, a more poorly differentiated histology, and mucinous and signet ring histology compared to older patients [7,33]. The rates of high microsatellite instability (MSI-H) are higher in EOCRC due to the higher incidence of Lynch syndrome. However, among the microsatellite-stable (MSS) cohort of metastatic CRC, patients with EOCRC have lower rates of *KRAS* and *BRAF* V600 mutations [31]. These observations suggest that the underlying biological process and basis of these clinicopathological conditions in EOCRC are different from those found in people above 50 years old.

The male gender is associated with a higher incidence of CRC compared to females. The incidence of rectal cancer is higher among young males compared to females. In 2019, the incidence rate of early-onset rectal cancer was 6.2 per 100,000 population among men, while it was 3.6 per 100,000 population among women. The higher proportion of men diagnosed with rectal cancer suggests a differential distribution of the tumor location between genders. Although the etiology between the gender differences is uncertain, it may be attributed to the varied exposure in dietary and lifestyle risk factors between both gender groups [34]. Some studies have explored the protective effect of estrogens and progestins against CRC in hormone replacement therapy in postmenopausal women [35,36].

The Chinese population in Singapore had the highest ASIR of CRC when compared to Indians and Malays. Previously, the Asia Pacific Consensus identified Chinese as an ethnic group that is more susceptible to CRC, and this was supported by cohort studies in Singapore and Malaysia [37-40]. In this study, although the incidence initially increased among the Chinese, the incidence rates have plateaued and declined over the past decade, except for early-onset rectal cancer, which continues to rise at 1.5% per year among young Chinese adults. However, the steep increase in the incidence of both colon and rectal cancers among the Malays across all age groups is concerning. This observation is supported by another study of age-period-cohort analyses in the SCR, stratified by ethnicity, that suggests a rising risk of CRC across all periods among the Malay population [41]. The rising incidence of EOCRC predominantly among Malays was also observed in Malaysia [42]. The varied epidemiologic trends across the 3 ethnic groups may be attributed to the differences in health-seeking behavior and lifestyle exposures between the groups in Singapore [41]. Modifiable risk factors, such as the rising rates and high prevalence of obesity among the Malays and the increased consumption of sweetened food, may play a role [43]. Malays were also less likely to participate in cancer screening compared to the Chinese. There is a need for tailored cancer-screening promotion campaigns to narrow the knowledge-behavior gap among the Malays [44]. Socioeconomic indicators, such as educational attainment, homeownership rates, and income levels, have shown improvement across all racial groups in Singapore [45]. Although there is a relatively similar socioeconomic environment in Singapore, the varying rates of the rise of EOCRC among the 3 races suggest that cultural, dietary, or genetic factors may potentially contribute to these differences. Further studies should be conducted that compare the lifestyle factors and social practices among the 3 ethnic groups and evaluate the impact on CRC incidence.

Our study suggests that patients with EOCRC have better cancer-specific survival compared to patients with CRC aged 65 years and older. However, there are conflicting data with regard to the survival outcomes of EOCRC compared to patients with late-onset CRC after adjustment for stage. A survival analysis of 35,713 patients with stage 3 colon cancer from the ACCENT database revealed that patients with EOCRC have improved overall survival, disease-free survival, and survival after recurrence. After adjusting for molecular markers (MMR, KRAS, BRAF), the prognostic impact of age of onset was lost [46]. However, there was a poorer 3-year relapse-free survival among patients with stage 3 EOCRC based on the pooled analyses of 16,349 patients from the International Duration Evaluation of Adjuvant Chemotherapy (IDEA) database. This was despite better treatment adherence and higher administered treatment intensity among the younger patients, hence suggestive of a more aggressive disease biology [47].

The current screening guidelines for CRC in Singapore start at 50 years of age for asymptomatic, average-risk individuals and have no impact on the incidence of early-onset CRC. Recently, the American Cancer Society recommended initiating CRC screening at age 45 years instead of 50 years for average-risk individuals to address the rising CRC incidence among young adults [48]. This recommendation was based on modeling data that suggest that this will prevent 29,400 CRC cases and 11,000 CRC deaths in the United States over the next 5 years [49]. Compared to the rising incidence of colon and rectal cancers in the United States, Europe, and Australia, our study suggests that rectal cancer among young adults is rising. Therefore, it is important for physicians to investigate young adults who present with anorectal symptoms, such as rectal bleeding, tenesmus, anal pain, and changes in bowel habits. Although these findings may suggest a need to re-evaluate the recommended age range for screening and consider extending screening to those 45-49 years of age, the incidence of EOCRC in Singapore remains low, and this strategy may not be cost effective. Despite the launch of the National Colorectal Screening Programme in 2011, the uptake of CRC screening remains low nationally at 41.7% [50,51]. However, flexible sigmoidoscopy among young adults is a less costly alternative as compared to colonoscopy and may capture the majority of EOCRCs since the tumor location of EOCRC is usually in the sigmoid colon or the rectum.

### Strengths and Limitations

Although this study provides a comprehensive overview of CRC trends in Singapore, further research into the biological basis of carcinogenesis among young adults and differences in modifiable risk factors among ethnic groups is warranted. Age-period-cohort analysis was limited due to missing population data of those aged 70 years and above. Additionally, the study was limited due to the lack of information about the proportion of hereditary cancer syndromes in the cohort. The prevalence of hereditary cancer syndromes among EOCRCs ranges from 5% to 35% compared to 2%-5% for CRCs overall [52-55]. Although genetic predisposition may be associated with EOCRC, the majority of EOCRCs are still sporadic. Additionally, screening of families with hereditary cancer syndromes may only capture a small proportion of EOCRCs [52]. Future research should focus on identification of risk factors and predictors of EOCRC to determine high-risk groups that should be targeted for screening before 50 years of age. It may be a more viable strategy to develop risk stratification tools that combine family history and blood-based tests to identify high-risk neoplasia among young adults.

### Conclusion

The incidence of rectal cancer among people under 50 years of age in Singapore appears to be increasing, especially among the Chinese and Malays. The differences in the epidemiologic trends among the 3 main ethnic groups may be due to varied lifestyle factors and social practices. Although it is possible that the observed increase may be attributable to lifestyle changes in the population over time, further studies are required to determine the underlying etiology. In contrast, CRC rates are decreasing among those above 65 years of age, and this may be due to the impact of the national screening program. These findings suggest the need for further research to diagnose CRC earlier among young adults and reduce the associated cancer-related morbidity and mortality.

## Appendix

Multimedia Appendix 1 High-resolution versions of Figures 2-9.

## Abbreviations

APC: annual percentage change
ASR: age-specific incidence rate
ASIR: age-standardized incidence rate
CRC: colorectal cancer
EOCRC: early-onset colorectal cancer
HR: hazard ratio
ICD: International Classification of Diseases
IRR: incidence rate ratio
SCR: Singapore Cancer Registry
SingStat: Department of Statistics Singapore
TNM: tumor, node, and metastasis

## Data Availability

Data collected from the Singapore Cancer Registry are not available publicly.
