# Supplementary material for: Trends in Early-Onset Colorectal Cancer in Singapore: Epidemiological Study of a Multiethnic Population
Source: JMIR Public Health Surveill. 2025 Feb 14;11:e62835. doi: 10.2196/62835 (PMC11888020; doi:10.2196/62835)
Supplement: Multimedia Appendix 1 [file publichealth_v11i1e62835_app1.pdf]

Multimedia Appendix 1: High-resolution versions of Figures 2-9

Figure 2: Joinpoint Regression of Age Standardized Incidence Rate (ASIR) of colorectal, colon and rectal cancer

Colorectal Cancer	Colon Cancer	Rectal Cancer
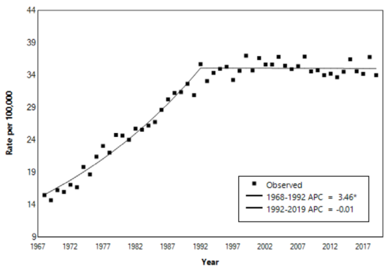	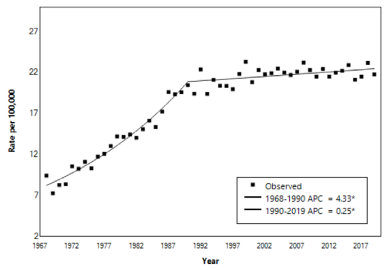	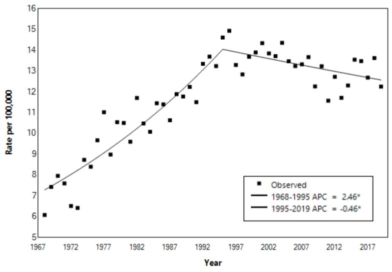

Figure 3: Joinpoint Regression of Age Specific Incidence Rate (ASR) of colorectal, colon and rectal cancer by age group & tumour location

	20 to 49 years	50 to 64 years	65 years and above
**Colorectal Cancer**	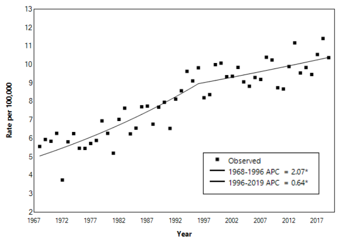	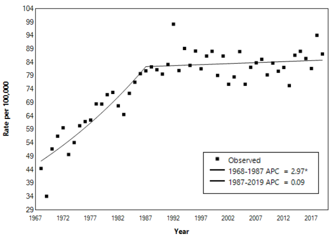	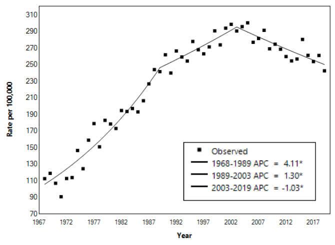
**Colon Cancer**	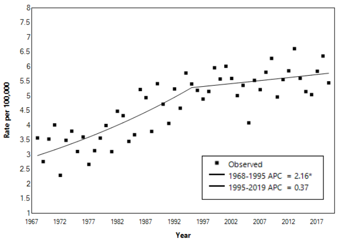	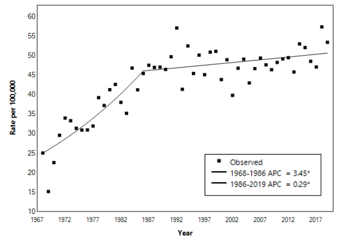	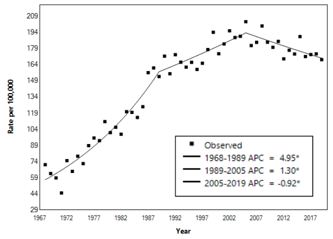
**Rectal Cancer**	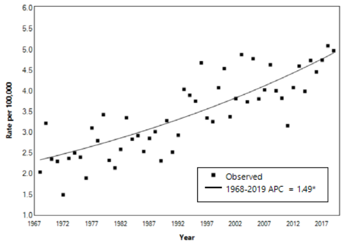	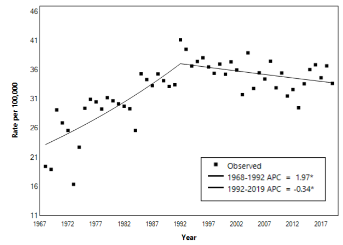	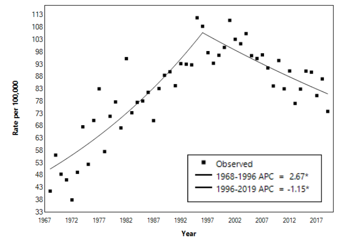

Figure 4: Joinpoint Regression of Age Standardized Incidence Rate (ASIR) of colorectal, colon and rectal cancer by gender (Male – Red, Female – Blue)

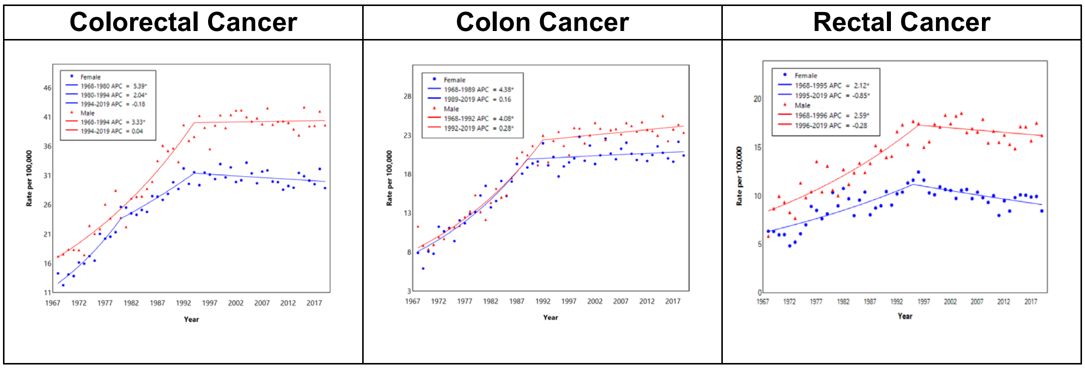

Figure 5: Joinpoint Regression of Age Specific Incidence Rate (ASR) of colorectal cancer, colon and rectal cancer by age groups and gender (Male – Red, Female – Blue)

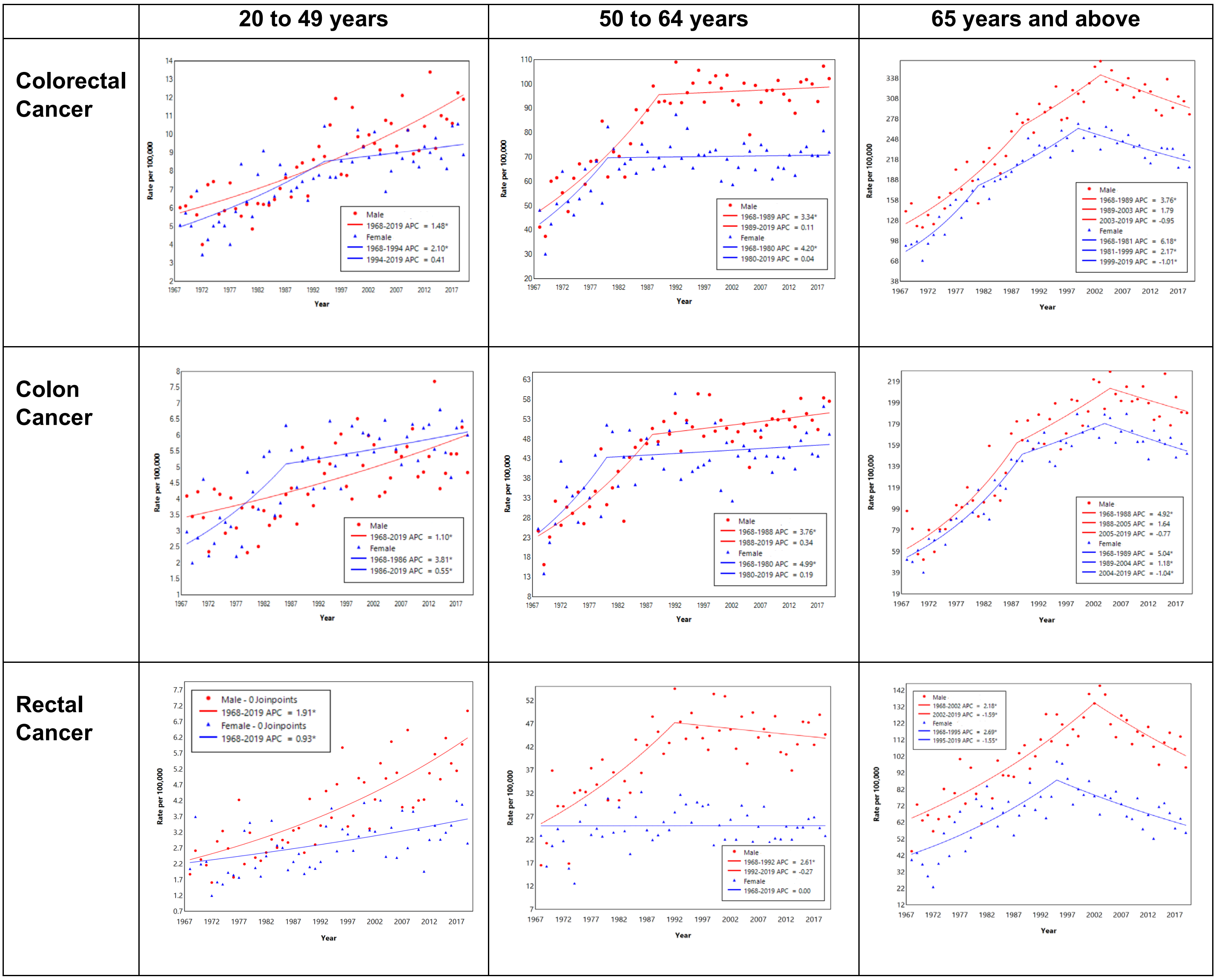

Figure 6: Joinpoint Regression of Age Standardized Incidence Rate (ASIR) of colorectal, colon and rectal cancer by ethnic groups (Chinese -- Blue, Indian -- Green, Malay -- Red)

Colorectal Cancer	Colon Cancer	Rectal Cancer
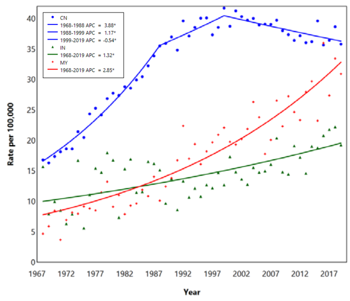	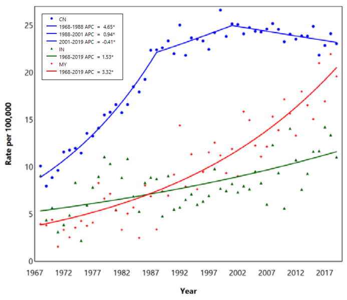	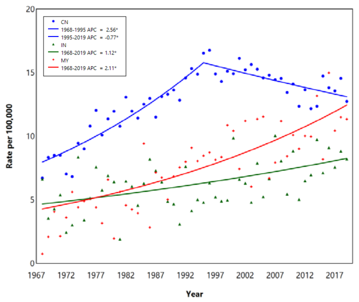

Figure 7: Joinpoint Regression of Age Specific Incidence Rate (ASR) of colorectal, colon and rectal cancer by age group, tumour location & ethnicity (Chinese/ CN-- Blue, Indian/ IN-- Green, Malay/ MY -- Red)

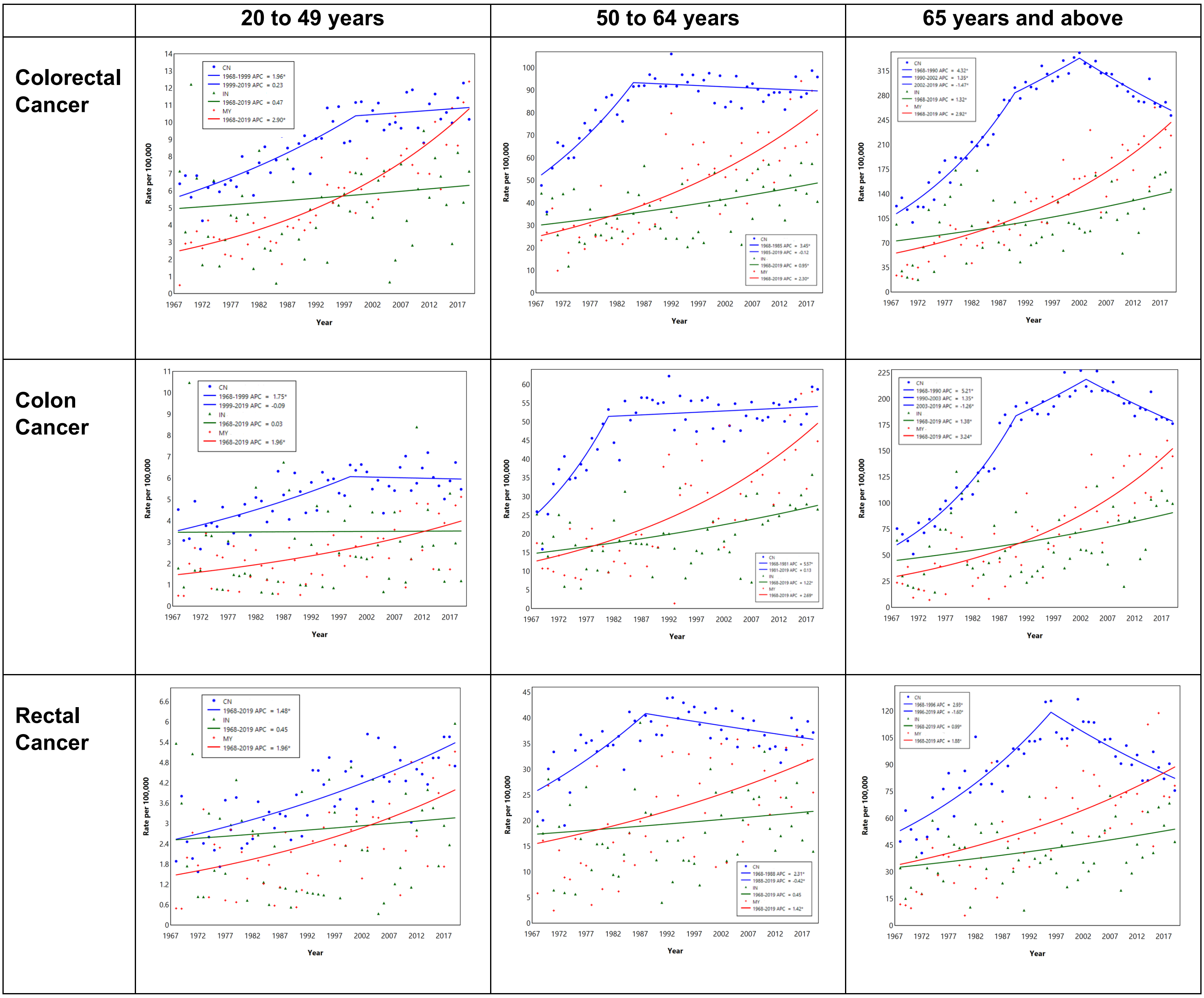

Figure 8: The incidence rate ratios (IRRs) by birth cohort for colorectal, colon and rectal cancers.

Colorectal Cancer	Colon Cancer	Rectal Cancer
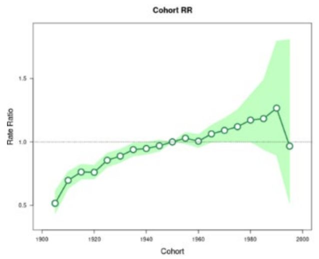	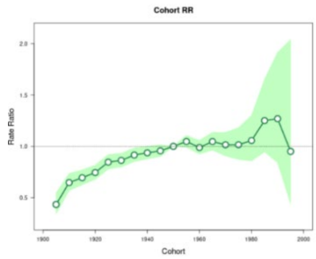	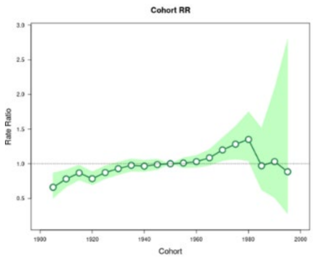

Figure 9: The age-specific APC (local drift) for colorectal, colon and rectal cancer

Colorectal Cancer	Colon Cancer	Rectal Cancer
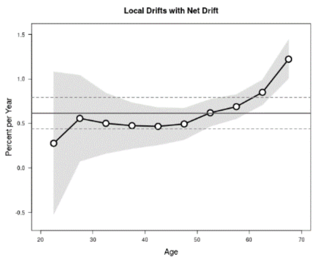	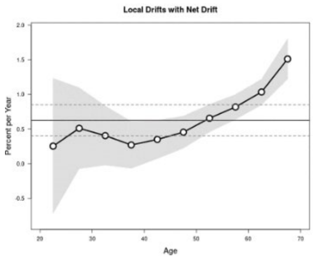	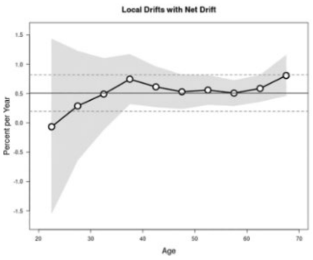
